# A randomised controlled trial of cognitive-behaviour therapy for clinical perfectionism: A preliminary study

**DOI:** 10.1016/j.brat.2006.12.003

**Published:** 2007-09

**Authors:** Caroline Riley, Michelle Lee, Zafra Cooper, Christopher G. Fairburn, Roz Shafran

**Affiliations:** Department of Psychiatry, Oxford University, Warneford Hospital, Oxford OX3 7JX, UK

**Keywords:** Cognitive-behaviour therapy, Clinical perfectionism, Randomised controlled trial

## Abstract

Perfectionism can be a problem in its own right and it can impede the progress of treatment of Axis I disorders. This study reports on a preliminary randomised controlled trial of cognitive-behaviour therapy (CBT) for “clinical perfectionism”. Twenty participants were randomly assigned to either immediate treatment (IT) (n=10) or a waitlist (NL) (n=10). Treatment consisted of ten sessions of CBT over eight weeks. Two participants did not complete the follow-up assessments (10%). Fifteen of the original 20 participants (75%) were clinically significantly improved after treatment and the effect size was large (1.8). Treatment gains were maintained at 8-week and 16-week follow-up.

## Introduction

Perfectionism can be dysfunctional in numerous ways. It is associated with various psychiatric disorders including eating disorders, obsessive compulsive disorder and depression (Flett & Hewitt, 2002), it can interfere with daily functioning and it can impede the progress of treatment of Axis I disorders (Blatt, Quinlan, Pilkonis, & Shea, 1995). Despite the clinical problems that can be associated with perfectionism, there is little agreement as to the nature of the construct. Some view dysfunctional perfectionism as a stable personality characteristic that is not readily amenable to change (Blatt et al., 1995). Others view it as a multidimensional construct with a strong interpersonal component (Hewitt & Flett, 1991; Hewitt, Flett, Besser, Sherry, & McGee, 2003), and still others argue that concern over mistakes are central (Frost, Marten, Lahart, & Rosenblate, 1990).

Most recently, we proposed a cognitive-behavioural model of a highly specific form of perfectionism, termed “clinical perfectionism”. This construct grew out of clinical observations made when treating patients with eating disorders, and refers specifically to a dysfunctional type of self-focused perfectionism in which the individual determinedly pursues self-imposed, personally demanding standards, despite adverse consequences. Central to this construct is the view that the individual's self-evaluation is largely, or even exclusively, dependent on the pursuit and achievement of their standards for performance (Shafran, Cooper, & Fairburn, 2002). A critical component of clinical perfectionism is not simply that the individual strives for high standards, but rather, the impact of not meeting these standards on self-evaluation. According to this cognitive-behavioural account, a range of maintaining mechanisms accounts for the persistence of clinical perfectionism including behaviour such as checking and avoidance, and cognitive factors such as dichotomous thinking operationalised as rigid rules (Riley & Shafran, 2005; Shafran et al., 2002). Such perfectionism is a problem in its own right, and is also suggested to impede successful treatment of Axis I disorders in cases where the domain of clinical perfectionism overlaps with that of the disorder (Shafran et al., 2002).

There has been some controversy regarding this construct (e.g., Hewitt et al., 2003; Shafran, Cooper, & Fairburn, 2003) and we are in full agreement that other forms of perfectionism such as socially oriented perfectionism can also pose clinical problems. Our goal in developing the specific construct of clinical perfectionism was to identify its maintaining mechanisms to enable the development of a highly focused brief intervention of clinical value. There has been some preliminary support for the construct and the cognitive-behavioural analysis. For example, a qualitative analysis was consistent with the conceptualisation (Riley & Shafran, 2005). Recently Dunkley, Blankstein, Masheb, and Grilo (2006) found that “self-criticism substantially accounts for the relation between perfectionism measures and depressive, anxiety and eating disorder symptoms” (p. 80) which is consistent with the view that self-evaluation is at the heart of perfectionism, rather than striving for high standards per se.

There is also support for the newly developed cognitive-behavioural intervention derived from the analysis. In a single case report of a patient with binge eating disorder, Shafran, Lee, and Fairburn (2004) found that the cognitive-behavioural intervention for clinical perfectionism was effective in the reduction of both clinical perfectionism and binge-eating and that improvements were maintained at five-month follow-up. Furthermore, a case-series study found that six out of nine participants showed some improvement in their perfectionism (Glover, Shafran, Brown, & Fairburn, in press).

These case studies contribute to the literature on the treatment of perfectionism that includes studies of emotion focused therapy (Greenberg & Bolger, 2001), psychodynamic therapy (e.g., Blatt, 1992), and cognitive-behavioural therapy (CBT) (e.g., Ferguson & Rodway, 1994; DiBartolo, Frost, Dixon, & Almodovar, 2001) including cognitive-behavioural self-help (Antony & Swinson, 1998). In one of the first randomised controlled trials of treatment for perfectionism, Pleva and Wade (in press) demonstrated that eight sessions of guided self-help, using the cognitive-behavioural manual by Antony and Swinson (1998), was effective in reducing perfectionism in a non-clinical sample. However, the newly developed cognitive-behavioural treatment specific for *clinical* perfectionism requires further evaluation using a rigorous design and clinical participants. Hence, the aim of the current study was to compare the therapeutic effects of CBT for clinical perfectionism with a waitlist (WL) control, and to investigate maintenance of any effects over eight and 16-weeks. It was hypothesised:

(1) CBT would be superior to a WL control condition.
(2) clinical perfectionism would be reduced after CBT, and these effects would be maintained at 8 week and 16-week follow-up.
(3) successful treatment of clinical perfectionism would result in an improvement in any accompanying Axis I diagnoses.

## Design

**Fig. 1:**
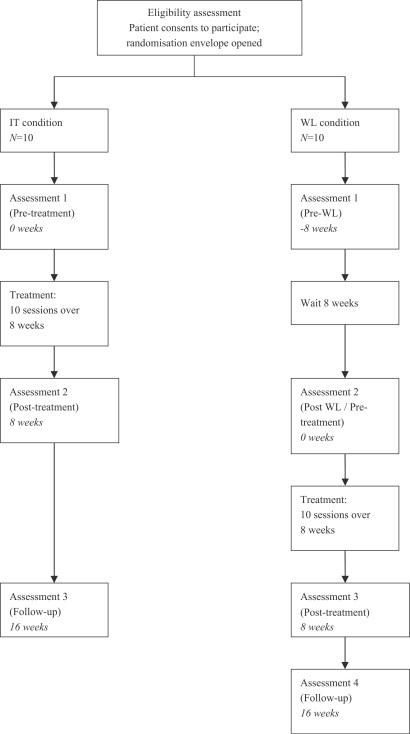
Study design.

Participants were randomly allocated to either immediate treatment (IT) or an initial eight-week WL group (n=10). After the initial eight-week delay, all those in the WL condition received the active intervention. Allocation was made using a random number list using a generated by computer, and concealing the result of each randomisation into numbered, sealed, opaque envelopes. These envelopes were only opened after the participant had consented to participate. Research assessments were conducted blind to the randomisation condition at pre-treatment, post-treatment, and on two follow-up occasions (8 and 16 weeks after treatment). Participants in the WL condition had one extra assessment at the beginning of the WL period^1^ (see Fig. 1). All assessments and therapy sessions took place at the Oxford University Department of Psychiatry and the study was approved by the NHS Central Office for Research Ethics Committee (COREC).

## Method

### Participants

Twenty participants (18 female) met inclusion criteria for clinical perfectionism according to a semi-structured interview based on the cognitive analysis. Fundamental to inclusion was that the individual's self-evaluation was largely, or even exclusively, dependent on the pursuit and achievement of their standards for performance and that their pursuit of these standards was interfering with daily functioning. Of these, 15 were referred by clinicians and five were recruited via advertisements.

### Measures

#### Clinical perfectionism

The Clinical Perfectionism Examination (CPE; Riley, Cooper, Fairburn, & Shafran, in preparation) is a 12-item investigator-led interview based on the cognitive-behavioural analysis (Shafran et al., 2002), designed to assess severity of clinical perfectionism. This interview has good test–retest reliability (*r*=.85), inter-rater reliability (*r*=.98) and internal consistency (α=.90). Convergent validity is adequate when compared with the Clinical Perfectionism Questionnaire (CPQ) (*r*=.57). The CPQ (Fairburn, Cooper, & Shafran, in preparation) is a twelve-item self-report questionnaire designed to assess current level of clinical perfectionism. Its individual items assess the cognitive, behavioural and affective components of setting goals and striving to meet them, and the consequences on the individual's self-evaluation when these standards are met or not met. Responses are rated on a four-point scale (1–4) ranging from “not at all” to “all of the time”. It has a four-week timeframe in order to be sensitive to clinical change. The measure is in the process of being validated.

#### Multidimensional perfectionism

Frost's Multidimensional Perfectionism Scale (MPS-F; Frost et al., 1990) is a 35-item self-report questionnaire that assesses “multidimensional perfectionism” using six subscales. Two of these subscales, “personal standards” and “concern over mistakes” were summed together for this study since the subscales both contain items consistent with the conceptualisation of clinical perfectionism. While the psychometric properties of using the measure in this way have not been assessed, the overall measure has good internal consistency (α=.77 to .93) and construct validity (*r*=.42 to .87). Hewitt and Flett's Multidimensional Perfectionism Scale (MPS-H; Hewitt & Flett, 1991) is a 45-item measure assessing multidimensional perfectionism using three subscales: “Self-Oriented Perfectionism”, “Other-Oriented Perfectionism” and “Socially Prescribed Perfectionism”. The MPS-H has good internal consistency (α=.82 to .87) and test–retest reliability (*r*=.75 to .88).

#### Associated psychopathology

The Structured Clinical Interview for DSM-IV-TR (SCID; First, Spitzer, Gibbon, & Williams, 2002) was used to identify the presence of a major depressive episode, eating disorder or anxiety disorder. The research assessor was formally trained in its use. The Beck Depression Inventory-II (BDI—II; Beck, Steer, & Brown, 1996) is a widely used 21-item self-report instrument used to measure the cognitive, behavioural and somatic severity of depression in adults and adolescents aged 13 and over. Its reliability and validity have been widely demonstrated and are summarised in the BDI—II manual (Beck et al., 1996). The Beck Anxiety Inventory (BAI; Beck, Epstein, Brown, & Steer, 1988) is a 21-item self report instrument used to assess severity of anxiety, and is especially developed to minimise its relationship with depression. The psychometric properties of this scale have been demonstrated in a variety of studies (Beck & Steer, 1990). The Brief Symptom Inventory (BSI; Derogatis & Melisaratos, 1983) is a 53-item self-report questionnaire assessing levels of psychopathology. The items describe a variety of difficulties and each is rated on a five-point scale, from “not at all” to “extremely”. The scale's authors report good internal consistency (α=.71 and .85) and test–retest reliability (*r*=.68 to .91).

All measures were administered by a research assessor who was blind to allocation.

### Treatment

The treatment was conducted by a postgraduate therapist who was trained in the treatment protocol (CR) and received weekly supervision from a senior Clinical Psychologist specialising in clinical perfectionism (RS). Treatment was conducted on an individual outpatient basis, and consisted of 10 sessions over eight weeks. Sessions were 50 min long and occurred twice weekly for the first three weeks, weekly for the subsequent three weeks and a two-week interval thereafter. The treatment was manualised, and the protocol consisted of four elements developed originally by Fairburn, Cooper, and Shafran (2003). These elements are: (1) identifying perfectionism as a problem and establishing maintaining mechanisms (e.g., repeated performance checking and avoidance, over-working or over-training); (2) conducting behavioural experiments to learn more about the nature of their perfectionism, and alternative ways of living (e.g., the impact of checking repeatedly vs. checking only occasionally); (3) psychoeducation and cognitive restructuring (in combination with behavioural experiments) to modify personal standards, self-criticism, “rules” and cognitive biases such as selective attention to perceived failure; (4) broadening the individual's scheme for self-evaluation, by examining existing methods of evaluating the self, and identifying and adopting alternative cognitions and behaviours (Fairburn, Marcus, & Wilson, 1993).

This protocol is one component of a new cognitive behavioural treatment for eating disorders (Fairburn et al., 2003) and was adapted specifically for this study.

### Treatment fidelity

Audiotapes of 20 therapy sessions (10% of all sessions) were randomly selected and rated by an independent clinical psychologist familiar with the treatment protocol. The rater listened to the tapes and rated the degree of adherence to the protocol as well as the quality of the therapy session, using the following dimensions: supportive encouragement, conveyance of expertise, and warmth. These dimensions were taken from criteria used by Agras, Walsh, Fairburn, Wilson and Kraemer (2000) in their study of treatment for bulimia nervosa. All ratings were made on a Likert scale ranging from 1 to 7. The mean score for adherence to the protocol was 5.35 (SD=1.18) i.e., good. Quality of therapy sessions was also good, with the mean score for supportive encouragement 5.65 (SD=.99), conveyance of expertise 6.00 (SD=1.21), and warmth 5.95 (SD=.95).

### Data analysis

Eighteen participants completed the full course of 10 sessions of treatment and attended all research assessments. An intent-to-treat analysis of the entire sample (Fergusson, Aaron, Guyatt, & Hébert, 2002) with the last data-point carried forward was carried out. Relevant MANOVAs and post hoc comparisons or planned contrasts were used to investigate the effects of CBT for clinical perfectionism immediately following treatment and at both follow-ups. The level of clinically significant change was calculated for the main outcome measure (the CPE) using Jacobson and Truax's (1991) definition and method. Since norms have not been established for the CPE and CPQ, participants were counted as “clinically significantly improved” if their post-treatment score was at least two standard deviations lower than the entire sample's mean pre-treatment score (Jacobson & Truax, 1991). There is no data on the scores of patients with clinically significant perfectionism as their primary difficulty on the MPS-F or the MPS-H and therefore clinically significant changes are not reported for these measures.

### Participants

The demographic characteristics and baseline scores of those assigned to IT, those in the WL control group, and all participants are shown in Table 1. There were no differences between those in the IT condition and the WL condition in terms of age [*t*(12.79)=−1.50, *p*<.05], or any of the measures [*F*(10,7)=1.167, *p*>.05]. The mean age of the entire sample was 29.90 years (*SD*=10.80) (Table 2).

## Results

### Hypothesis 1: CBT for clinical perfectionism will be superior to a WL control condition

**Fig. 2:**
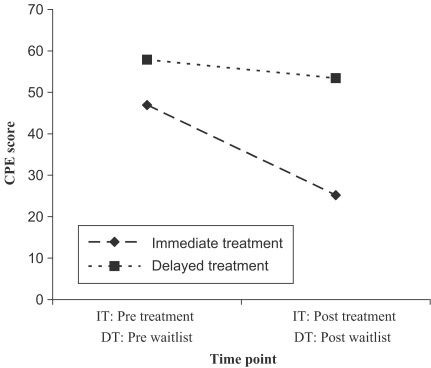
Clinical Perfectionism Examination scores for those receiving immediate treatment (IT) and delayed treatment (DT) before and after treatment (outlier removed).

A MANOVA indicated that changes in the two measures of clinical perfectionism (the CPE and the CPQ) were significantly greater in those in the IT group than the WL group [*F*(1, 18)=15.92, *p*<.005) (Fig. 2). Paired samples *t*-tests were conducted to compare change scores across the two groups for the CPE and the CPQ separately. These indicated that change scores were significantly greater for those in the IT group as compared with the WL group for the CPE [*t*(18)=4.04, *p*<.005] but not the CPQ [*t*(18)=1.75, *p*>.05]. However, when an outlier (the change score was over two standard deviations away from the mean) was removed,^2^ change scores on the CPQ were also statistically different: [*t*(17)=3.14, *p*<.01]. The effect sizes for the WL group were small: *d*=0.27 for the CPE and *d*=0.38 for the CPQ. Effect sizes for the IT group were both large: *d*=2.05 for the CPE and *d*=1.36 for the CPQ (Cohen, 1988). There were no statistically significant differences between the two groups on any of the subscales of the MPS-F or the MPS-H [*F*(6,13)=1.51, *p*>.05].

### Hypothesis 2: Clinical perfectionism will be reduced after CBT, and these effects will be maintained at eight and 16 week follow-up

The mean scores for the entire sample pre- and post-treatment and at both follow-up assessments are shown in Table 3.

#### Clinical perfectionism measures

There was a significant main effect of time on the CPE and the CPQ [*F*(3,15)=14.79, *p*<.001] indicating that scores changed over the course of the study. A repeated measures ANOVA revealed a significant difference in CPE scores [*F*(3,15)=13.89, *p*<.001]. A paired samples *t*-tests showed that scores at post-treatment were significantly lower than at baseline [*t*(19)=6.69, *p*<.001]. Fifteen of the 20 participants were clinically significantly improved (Jacobson & Truax, 1991) at post-test and the effect size was large: *d*=1.83. Scores at eight week and 16-week follow-up were also significantly lower than at baseline [*t*(19)=6.41, *p*<.001; *t*(17)=5.51, *p*<.001, respectively], indicating that these gains were maintained over time. No significant change was noted from post-treatment scores to either follow up assessment (*p*>.05). The same analyses revealed significant differences in CPQ scores across the four time points [*F*(3,15)=8.64, *p*=.001] with scores at post-treatment significantly lower than scores at baseline [*t*(19)=5.61, *p*<.001]. Similarly, scores at both eight and 16-week follow-up were also significantly lower than scores at baseline [*t*(18)=5.46, *p*<.001; *t*(17)=4.45, *p*<.001, respectively] indicating that these changes were maintained over time. No significant change was noted from post-treatment scores to either follow up assessment (*p*>.05). The effect size for pre- and post-treatment changes in CPQ scores was large: *d*=1.31 (Cohen, 1988).

#### Other measures of perfectionism

There was a significant main effect of time for the other perfectionism measures [*F*(3,15)=14.77, *p*<.001]. Specifically there was a significant difference in the summed subscales of the MPS—F [*F*(3,15)=7.60, *p*<.005] over time, and a paired samples *t*-test showed that scores at post-treatment were significantly lower than at baseline [*t*(19)=2.67, *p*<.05]. Scores at eight week and 16-week follow-up were also significantly lower than at baseline [*t*(19)=5.10, *p*<.001 and *t*(17)=3.73, *p*<.005, respectively]. Scores at the first and second follow up assessments did not change from post-treatment scores (*t*(19)=1.83, ns and *t*(17)=0.37, ns respectively). The same pattern of findings was obtained for the self-oriented perfectionism subscale of the MPS—H [*F*(3,15)=6.58, *p*=.005] with scores at post-treatment were significantly lower than at baseline [*t*(19)=4.13, *p*=.001], and these gains were maintained at eight week follow up [*t*(19)=4.83, *p*<.001], and 16-week follow-up [*t*(17)=4.18, *p*=.001]. Scores at the first and second follow up assessments did not differ from post-treatment scores (*t*(19)=0.99, ns and *t*(17)=0.77, ns respectively). For the OOP and SPP scores, there was a significant difference across time (*F*(3,15)=9.41, *p*=.001 and *F*(3,15)= 7.86, *p*<.005, respectively) and scores at post-treatment and eight week follow up significantly lower than at baseline (all *p*<.05); these gains were not maintained at 16-week follow up [ *p*>.05].

### Hypothesis 3: Treatment of clinical perfectionism will result in an improvement in Axis I diagnoses

A repeated measures MANOVA was conducted to investigate the effects of CBT for clinical perfectionism on associated psychopathology i.e., BDI, BAI, and BSI scores. Across all measures, there was a significant main effect of time [*F*(3,12)=4.83, *p*=.02]. A one-way repeated measures ANOVA indicated that BDI scores reduced significantly across time [*F*(3,14)=3.63, *p*=.04]. Specifically, compared with baseline, scores were reduced post treatment and at eight week follow up [*t*(19)=3.34, *p*=.003 and *t*(18)=2.76, *p*=.013, respectively] but not at 16-week follow up [*t*(16)=2.04, *p*>.05]. The same pattern of findings was obtained for the BAI and BSI [*p*<.05] with reductions at post treatment and also at eight week although the gains were also maintained at 16 follow-up [*p*<.05].

Participants were interviewed using the SCID (First et al., 2002) before and after treatment. Prior to treatment, 10 (50%) met criteria for an anxiety disorder or major depressive episode (6 generalised anxiety disorder; 5 social phobia; 2 obsessive-compulsive disorder; 2 major depressive episode; 1 panic disorder with agoraphobia; 1 specific phobia). Immediately following treatment, five participants (25%) met criteria (one of whom had not met criteria pre-treatment) reducing to four participants at eight-week and 16-week follow up.^3^

Participants who were randomised to the WL condition were also interviewed with the SCID prior to entering the WL phase. Six participants met criteria for an anxiety disorder or major depressive episode at this stage and this did not change when they were reassessed at the end of the WL. To further investigate the effect of treatment vs. WL across time on BDI, BAI and BSI scores, a 3 (time; 0 vs 8 vs 16-weeks)×2 (condition; IT vs DT) mixed MANOVA was carried out. This indicated a significant time by condition interaction (*F*(2,14)=5.29, *p*<.05). Further analyses (using paired samples *t*-tests for each condition) indicated that for those receiving immediate treatment, BDI and BSI scores reduced significantly between the start and end of treatment (*t*(9)=4.68, *p*=.001 and *t*(9)=3.16, *p*<.05, respectively), whereas there was no significant change for those in the waiting list condition between the start and end of their WL period, either for BDI or BSI scores (*t*(8)=0.17, *p*>.05 and *t*(8)=0.64, *p*>.05, respectively].

## Discussion

This study provides preliminary evidence that a brief, cognitive-behavioural intervention is effective in reducing clinical perfectionism, and is superior to an eight-week WL condition on measures of clinical perfectionism. In this small study, effect sizes were large (comparing well with trials of CBT for other forms of psychopathology, e.g., Chambless & Gillis, 1993) and reductions in clinical perfectionism were maintained at eight week and 16-week follow-up. Fifteen participants were clinically significantly improved at post-treatment assessment and remained so at both follow-up assessments. The number of participants meeting diagnostic criteria for an anxiety disorder or major depressive episode halved after treatment which is reflected in the reductions in self-report measures of psychopathology, and comparisons of those receiving immediate vs delayed treatment tentatively suggest that the intervention had a clinically significant impact on co-occurring psychopathology.

A variety of measures of perfectionism were used in this study. The scores on both Multidimensional Perfectionism Scales significantly reduced with treatment, although the changes on the two interpersonal subscales of the MPS-H did not endure and the changes were not significantly different in the subset receiving immediate treatment compared to the WL group. This could be for a variety of reasons, including the small sample size in each group and the lack of time-frame specified by the multidimensional measures. The multidimensional measures purposefully do not specify a time-frame because the construct that they are designed to assess is “trait-like”, stable and enduring (e.g., Cox & Enns, 2003). By contrast, clinical perfectionism is not viewed as a stable dysfunctional personality style but rather a form of psychopathology maintained by specific cognitions and behaviour. It is not intended that this brief, focused intervention reduces other aspects of perfectionism. It also remains to be established whether the decreases in clinical perfectionism are merely due to changes in other psychopathology. This is likely to be the case for some patients since perfectionism has been shown to change with successful treatment of an Axis I disorder (e.g. Lundh & Öst, 2001; Rosser, Issakidis, & Peters, 2003). However, it is also likely that there are some patients for whom clinical perfectionism is a barrier to change in the Axis I psychopathology and for whom a specific intervention is warranted.

There are a number of limitations of the current study that should be taken into account. First, and foremost, there was a small sample size comprised predominantly of women. Although a significant difference between the WL controls and the group treated with CBT on the measures of clinical perfectionism were found, the study may have been underpowered to detect smaller differences in other measures. Second, although the study controlled for the passage of time and the effects of repeated assessment by using a WL comparison group, the study lacked a comparison group to control for non-specific effects. Thus, it cannot be concluded that changes were specific to the intervention. Third, research is needed to establish whether the intervention has any impact on other aspects of perfectionism, including perfectionistic vulnerability. For example, correlational analyses would have allowed further examination of whether or not treatment reduced participants’ underlying vulnerability to engaging in perfectionist beliefs and behaviours. Finally, the study only assessed anxiety disorders and major depressive episodes and did not assess the degree of impairment to daily functioning caused by clinical perfectionism, or accompanying Axis I disorders, before treatment or afterwards. Including a measure of daily functioning would have supported clinical judgment that participants’ clinical perfectionism was causing a significant clinical problem as well as providing a further indication of the effectiveness of the intervention.

In conclusion, this small randomised controlled trial provides preliminary data that a specific form of perfectionism (clinical perfectionism) that interferes with patient's functioning, can be reduced with a focused, brief intervention based on a clearly specified theory. Acceptability of the treatment was good with only one participant dropping out of treatment and an additional participant declining to complete the follow-up assessment. Given that the intervention is cognitive-behavioural and relatively brief, it could be used as an adjunct to existing cognitive-behavioural interventions when clinical perfectionism is proving to be a barrier to change, or as a stand-alone treatment for people with clinical perfectionism as their primary problem.

## Figures and Tables

**Table 1:** Participant information (pre treatment or pre waitlist)

Measure	Waitlist group (n=10)	Immediate treatment group (n=10)	Entire sample (n=20)
Age	33.40 (13.39)^a^	26.40 (6.29)^a^	29.90 (10.80)
Marital status	60% single	40% single	50% single
Occupation	5 students, 1 employed, 4 not working	4 students, 5 employed, 1 not working	9 students, 6 employed, 5 not working
Axis I disorder	8 with Axis I diagnoses	6 with Axis I diagnoses	6 without Axis I diagnoses
			9 with 1 Axis 1 diagnosis
			5 with 2+ Axis 1 diagnosis
*Clinical perfectionism*			
CPE	57.89 (6.23)^a^	46.90 (10.58)^a^	52.11 (10.24)
CPQ	35.33 (5.96)^a^	35.70 (5.07)^a^	35.52 (5.36)
*Multidimensional perfectionism*			
MPS-F	102.33 (16.97)^a^	101.2 (18.59)^a^	101.74 (17.35)
MPS-F CM & PS	65.56 (9.42)^a^	63.50 (10.48)^a^	64.47 (9.77)
MPS-H SOP	94.44 (8.66)^a^	86.50 (16.65)^a^	90.26 (13.73)
MPS-H OOP	58.67 (14.00)^a^	62.40 (22.78)^a^	60.63 (18.72)
MPS-H SPP	67.22 (16.03)^a^	66.80 (15.22)^a^	67.00 (15.36)
*Associated psychopathology*			
BDI	24.44 (11.67)^a^	24.90 (10.03)^a^	24.68 (10.53)
BAI	17.88 (8.70)^a^	16.00 (7.42)^a^	16.94 (7.90)
BSI	86.67 (42.60)^a^	70.20 (35.10)^a^	78.00 (38.65)

Means with different superscripts are significantly different at the *p*<.05 level.

**Table 2:** Mean scores for waitlist and immediate treatment conditions (outlier removed)

Measure	Waitlist group			Immediate treatment group		
	Pre-waitlist	Post-waitlist	Change score	Pre-treatment	Post-treatment	Change score
Age	33.40 (13.39)^a^			26.40 (6.29)^a^		
*Clinical perfectionism*						
CPE	57.89 (6.23)	53.44 (12.13)	4.45 (7.30)^a^	46.90 (10.58)	25.20 (13.15)	21.70 (12.12)^b^
CPQ	35.33 (5.96)	34.89 (4.78)	.44 (2.92)^a^	35.70 (5.07)	28.80 (6.25)	7.40 (6.02)^b^
*Multidimensional perfectionism*						
MPS-F	102.33 (16.97)	101.00 (13.28)	1.33 (9.02)^a^	101.20 (18.59)	91.10 (25.98)	10.10 (18.95)^a^
MPS-F CM & PS	65.56 (9.42)	65.33 (6.95)	.02 (4.64)^a^	63.50 (10.48)	55.80 (9.50)	7.70 (12.37)^a^
MPS-H SOP	94.44 (8.66)	92.88 (8.80)	1.56 (7.21)^a^	86.50 (16.65)	75.20 (15.29)	11.30 (16.31)^a^
MPS-H OOP	58.67 (14.00)	59.22 (12.99)	−.55 (9.05)^a^	62.40 (22.78)	53.70 (15.03)	8.70 (13.82)^a^
MPS-H SPP	67.22 (16.03)	67.00 (14.27)	.22 (5.50)^a^	66.80 (15.22)	59.50 (12.66)	7.30 (16.08)^a^
*Associated psychopathology*						
BDI	24.44 (11.67)	21.90 (13.19)	.33 (5.94)^a^	24.90 (10.03)	14.40 (15.28)	10.50 (7.09)^b^
BAI	17.88 (8.70)	17.50 (8.17)	.22 (6.74)^a^	16.00 (7.42)	14.50 (11.17)	3.33 (11.16)^a^
BSI	86.67 (42.60)	77.40 (36.44)	3.89 (18.13)^a^	70.20 (35.10)	52.40 (44.58)	17.80 (17.79)^b^

Means with different superscripts are significantly different at the *p*<.05 level.

**Table 3:** Mean outcome scores (N=20)

Measure	Pre-treatment	Post-treatment	8-week follow-up	16-week follow-up
*Clinical perfectionism*				
CPE	48.85 (12.33)^a^	26.25 (15.81)^b^	24.05 (17.40)^b^	22.00 (19.10)^b^
CPQ	34.75 (5.33)^a^	27.75 (5.97)^b^	25.26 (5.19)^b^	25.56 (6.11)^b^
*Multidimensional perfectionism*				
MPS-F	100.35 (16.20)^a^	86.80 (21.20)^b^	81.55 (19.30)^b^	84.39 (20.54)^b^
MPS-F CM & PS	64.00 (9.73)^a^	53.05 (10.57)^b^	50.00 (11.48)^b^	52.59 (14.40)^b^
MPS-H SOP	88.20 (14.46)^a^	73.30 (16.14)^b^	70.95 (15.39) ^b^	69.06 (18.03)^b^
MPS-H OOP	60.40 (18.01)^a^	49.05 (14.76)^b^	52.10 (12.53)^b^	54.72 (12.69)^a,b^
MPS-H SPP	66.60 (14.04) ^a^	56.45 (12.98)^b^	55.05 (14.08)^b^	57.44 (17.39)^a,b^
*Associated psychopathology*				
BDI	23.40 (11.51)^a^	14.50 (13.12)^b^	15.94 (15.08)^a^	15.71 (15.73)^a,b^
BAI	16.79 (7.64)^a^	12.20 (8.79)^b^	11.21 (8.63)^b^	10.11 (10.58)^b^
BSI	73.80 (35.02)^a^	48.10 (34.87)^b^	42.40 (38.40)^b^	35.88 (35.09)^b^

Means with different superscripts are significantly different at the *p*<.05 level.

## Footnotes

1 This design meant that for the final 16 week assessment for these individuals, the assessor was not blind to whether they had originally been in the WL condition.

2 This participant's change score on the CPQ was 20 compared with a mean change score of .44 for this group. It is likely that she completed the questionnaire incorrectly at her initial assessment.

3 Only 18/20 participants completed the eight and 16 week follow-up assessments. Of the two participants who did not, one met criteria for a major depressive disorder at post-treatment. The other participant was considered to have bipolar disorder (although not formally diagnosed with the SCID) that was unaffected by the intervention.
